# Lim homeobox genes in the Ctenophore *Mnemiopsis leidyi: *the evolution of neural cell type specification

**DOI:** 10.1186/2041-9139-3-2

**Published:** 2012-01-13

**Authors:** David K Simmons, Kevin Pang, Mark Q Martindale

**Affiliations:** 1 Kewalo Marine Laboratory, Department of Zoology, University of Hawaii at Manoa, Honolulu, HI, USA, 96813; 2 Sars, International Centre for Marine Molecular Biology, University of Bergen, Thormøhlensgate 55, 5008 Bergen, Norway

## Abstract

**Background:**

Nervous systems are thought to be important to the evolutionary success and diversification of metazoans, yet little is known about the origin of simple nervous systems at the base of the animal tree. Recent data suggest that ctenophores, a group of macroscopic pelagic marine invertebrates, are the most ancient group of animals that possess a definitive nervous system consisting of a distributed nerve net and an apical statocyst. This study reports on details of the evolution of the neural cell type specifying transcription factor family of LIM homeobox containing genes (Lhx), which have highly conserved functions in neural specification in bilaterian animals.

**Results:**

Using next generation sequencing, the first draft of the genome of the ctenophore *Mnemiopsis leidyi *has been generated. The Lhx genes in all animals are represented by seven subfamilies (*Lhx1/5, Lhx3/4, Lmx, Islet, Lhx2/9, Lhx6/8*, and *LMO*) of which four were found to be represented in the ctenophore lineage (*Lhx1/5, Lhx3/4, Lmx*, and *Islet*). Interestingly, the ctenophore Lhx gene complement is more similar to the sponge complement (sponges do not possess neurons) than to either the cnidarian-bilaterian or placozoan Lhx complements. Using whole mount *in situ *hybridization, the Lhx gene expression patterns were examined and found to be expressed around the blastopore and in cells that give rise to the apical organ and putative neural sensory cells.

**Conclusion:**

This research gives us a first look at neural cell type specification in the ctenophore *M. leidyi*. Within *M. leidyi*, Lhx genes are expressed in overlapping domains within proposed neural cellular and sensory cell territories. These data suggest that Lhx genes likely played a conserved role in the patterning of sensory cells in the ancestor of sponges and ctenophores, and may provide a link to the expression of Lhx orthologs in sponge larval photoreceptive cells. Lhx genes were later co-opted into patterning more diversified complements of neural and non-neural cell types in later evolving animals.

## Background

LIM Homeobox (Lhx) genes were first isolated from the nematode, *Caenorhabditis elegans*, where the Lhx homolog, *MEC-3*, was shown to be required for the proper differentiation of touch receptor neurons [1]. Subsequent studies in *C. elegans *and rat isolated *LIN-11 *and *Islet1*, respectively, which together with *MEC-3 *are the founding members for the LIM family acronym [2,3]. Phylogenetically, Lhx genes were originally subdivided into six subfamilies, *Lhx1/5, Lhx2/9, Lhx3/4, Lhx6/8, Islet*, and *Lmx *[4]. Lhx proteins are composed of tandem zinc-finger LIM domains at the N-terminus, which function by binding specific co-factors that mediate their function, while the helix-turn-helix homeodomain (HD) interacts with DNA in a sequence-specific manner [5]. While LIM domains and homeodomains are found in non-metazoan eukaryotes, the specific combination of LIM-LIM-HD is only found in animals [6].

Lhx genes have roles in cell specification, tissue differentiation and neural patterning. In both vertebrate and invertebrate taxa, Lhx genes have conserved roles in the patterning of sensory neurons, interneurons, and motor neurons (reviewed in [4,7]). It has been suggested that cells expressing different combinations of Lhx genes form a "LIM code" that is important in specifying cell types within a tissue or organ [8], and motor neuron axon pathway finding [5].

The *C. elegans Lhx1/5 *genes, *LIN-11 *and *MEC-3*, are required for the terminal differentiation of non-overlapping sensory, motor neurons and interneurons [1,9,10]. Mouse null mutants of the *Lhx1 *family die during mid-gastrulation due to massive head defects [11], while the *Lhx5 *homologue is expressed in the anterior neural plate and parts of the developing diencephalon [12]. At later stages, the expression pattern of *Lhx5 *extends to parts of the midbrain, hindbrain and spinal cord [13]. A study (reviewed in [5] on the LIM code for axon path finding in motor neuron subtypes revealed that depending on the combination of Lhx genes expressed in motor neurons, different motor neuron subtypes are produced. *Lhx1 *when expressed with *Islet2 *produces motor neurons that project into ventral limb bud musculature, while *Islet1, Islet2*, and *Lhx3 *expressing motor neurons project into the medial motor column, and different motor neurons are produced when *Isl1 *and *Isl2 *are expressed either together or separately. In addition to neural patterning, *Lhx1 *is also known for its role in blastoporal organizer activity during gastrulation in *Xenopus*, ascidians, amphioxus, and cnidarians [14] as well as endoderm specification in ascidians [15], amphioxus [16] and mice [17].

The *Lhx2/9 *group, also known as the *apterous *group (named after the *Drosophila *gene), has diverse roles in patterning the nervous system, wing development, muscle development, axon guidance, and neurotransmitter choice [18,19]. The *C. elegans Lhx2/9 *homologue *ttx-3 *is expressed in a pair of interneurons and anterior muscle cells that project into the nerve ring [20]. The vertebrate *Lhx2/9 *homologues are expressed in the nerve cords, eyes, olfactory organs and limbs [4].

The *Lhx3/4 *subgroup expression patterns were found to be restricted to post mitotic neurons in *Drosophila*. The function of *Lhx3/4 *genes in *Drosophila *is in axon guidance of specific motor neurons when combined with the expression of Lhx gene *islet *[21]. In the developing chick embryo, *Lhx3/4 *has been shown to specify the formation of interneurons in the presence of one of its binding partners *Ldb1 *(LIM domain-binding protein 1), while additional expression within the same cells with the Lhx gene *Islet1 *specifies the formation of motor neurons [22], which show that Lhx genes function in a combinatorial fashion called the 'LIM code' to specify specific neural identities [4,8,23].

The *Drosophila Lhx6/8 *homologue arrowhead is expressed in the nervous system and has yet to be functionally analyzed [24]. The *C. elegans *homologue is expressed in sensory, motor and interneurons in the brain [25]. The vertebrate members of this subfamily are expressed in the forebrain and first branchial arch [26].

The *islet *gene subfamily is expressed in mesodermal derived cells and in subsets of motor and interneurons of the central nervous system (CNS) of *Drosophila *[27]. In vertebrates, *Islet *paralogs are expressed in many tissues, including heart, liver, pancreas, brain, and eyes [28-30].

The *C. elegans *homologue to the group *Lmx *is expressed in post-mitotic neurons controlling axon guidance and the synthesis of the neurotransmitter GABA. It is also expressed in endothelial cells of the uterus and in the excretory system [31]. The vertebrate chick homologue *Lmx-1b *is involved in dorsal ventral patterning of limbs and in patterning the otic vesicle [32].

**Figure 1:**
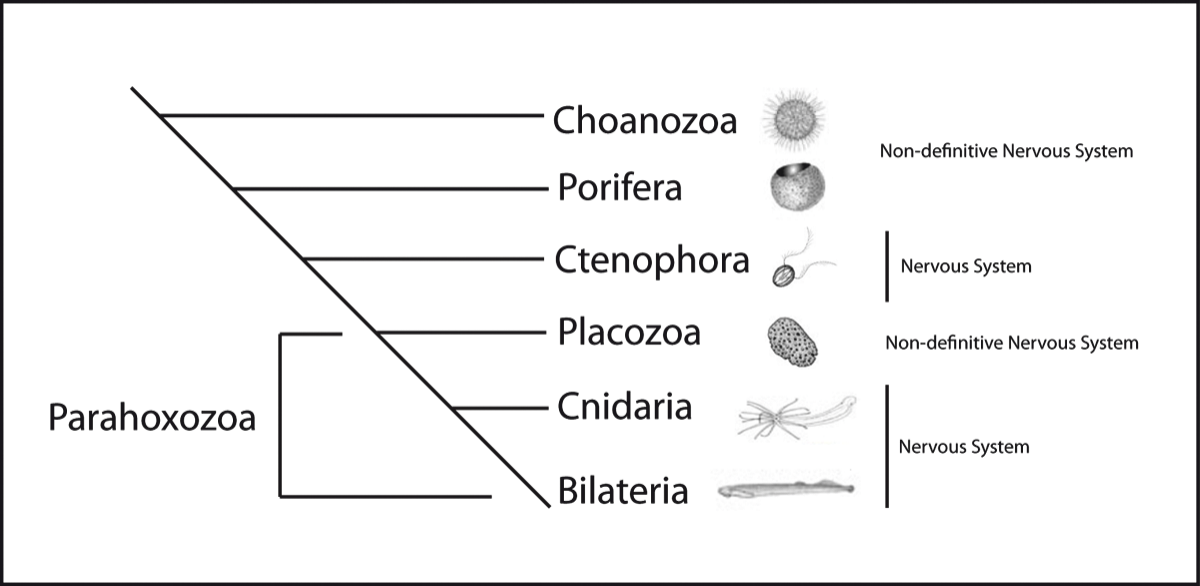
**Phylogenetic relationships of 'basal metazoa'**. Phylogenetic relationships of early branching metazoan taxa, based on data from recent studies [45-47]. Non-definitive nervous systems refer to the lack of morphological and physiological data to confirm their existence.

Novel neural cell types arose at some point during the evolution of metazoans, as nervous systems evolved and diversified throughout the bilaterian radiation. Nested between single-celled choanoflagellate-like ancestors (Figure 1) and the Bilaterian radiation of metazoans are four groups: Porifera, Ctenophora, Cnidaria, and Placozoa. While ctenophores and cnidarians have distinct nerve cells and other neural structures, poriferans and placozoans do not [33,34]. Interestingly, the genomes of the sponge, *Amphimedon queenslandica*, and the placozoan, *Trichoplax adhaerens*, have revealed the presence of most of the genes involved in forming the post-synaptic scaffold, as well as neurotransmitter biosynthesis [33-35]. Even choanoflagellates, the closest extant sister group of the Metazoa, possess genes for many of the post-synaptic scaffolding proteins, showing that many of the components to build a neuron likely predated its evolution [36]. Thus, it is of some interest to determine how the molecular components were assembled in early branching metazoans to form functionally integrated nervous systems.

Cnidarians are now well accepted to be the sister group to all bilaterians [37-41]. The anthozoan cnidarian, *Nematostella vectensis*, possesses members of all six Lhx subfamilies, as does the placozoan, *Trichoplax*, suggesting that complete diversification already occurred in the ParaHoxozoan common ancestor [6]. The sponge, *Amphimedon*, only has members of three subfamilies: *Lhx1/5, Lhx3/4*, and *Islet. Amphimedon Lhx1/5 *and *Lhx3/4 *were found to be expressed within putative photosensory cells surrounding larval pigment cells, while Islet is ubiquitously expressed [6]. In *Trichoplax*, all Lhx genes were shown to be expressed in adult animals through the use of RT-PCR; however, spatio-temporal patterns were not examined [6]. Expression patterns of *NvLhx6/8, NvLhx1/5*, and *NvLmx *in *Nematostella *overlap with previously described neural territories [6]. Interestingly, while the *Amphimedon *and *Nematostella *Lhx genes are all on separate genomic scaffolds, a single *Trichoplax *scaffold contains a cluster of *Lmx, Lhx3/4, Lhx6/8*, a *LIM-only *gene, and *Lhx2/9 *further downstream, suggesting that the Lhx family arose via tandem duplications [6].

Ctenophores occupy a highly debated phylogenetic position, once grouped with cnidarians forming the group Coelenterata [42,43]. Ctenophores are now thought to have diverged prior to cnidarians and may actually be the earliest extant metazoan phyla that possess definitive neurons [37,38]. Comparisons of gene complements between the four basal metazoans will be helpful in giving support for one or more of the various competing phylogenetic hypotheses (reviewed in [44]). Recent gene content studies [45-47] give consistent support for Porifera and Ctenophora diverging prior to the ParaHoxozoa (Placozoa, Cnidaria, Bilateria), however identifying the earliest branching taxon remains problematic.

For this study the genomic complement of Lhx genes was examined in the lobate ctenophore *M. leidyi*. Using the recently sequenced genome of *M. leidyi*, four Lhx genes were predicted by preliminary searches [45]. We further analyzed these genes by obtaining their full sequences through RACE PCR. Using whole mount *in situ *hybridization, we examined the expression patterns of the four Lhx genes throughout development. These genes are expressed in discreet sensory cell types, and in an overlapping fashion within the apical sensory organ, a highly innervated nervous structure.

## Results

### Gene identification, structure and genomic organization

**Figure 2:**
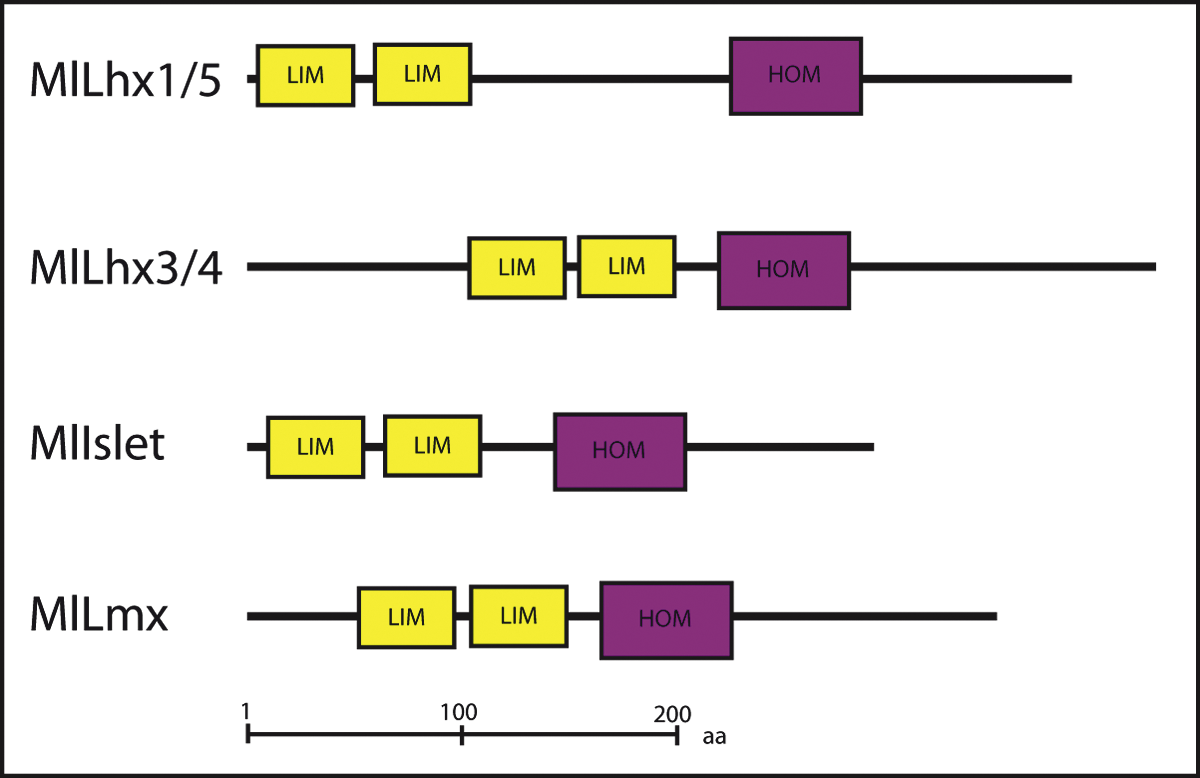
**Domain structure of *Mnemiopsis *Lhx genes**. The domain structure of the *Mnemiopsis leidyi *Lhx genomic complement was predicted by using the SMART database [48]. Yellow boxes indicate the tandem LIM zinc finger binding domains, pink boxes indicate the homeodomain sequence.

Partial sequence information from the four *M. leidyi *Lhx homeobox genes previously reported [45] were used to design non-degenerate primers. Full-length cDNA transcripts for all four *Mnemiopsis *Lhx genes were isolated by 5' and 3' RACE RT-PCR from mixed-stage embryonic cDNA. The four sequences have been submitted to GenBank: *MlLhx1/5 *[Genbank: JF912807], *MlLhx3/4 *[Genbank: JF912808], *MlIslet *[Genbank: JF912806], and *MlLmx *[Genbank: JF912809]. All of the Lhx genes isolated contained two tandem N-terminal LIM domains followed by a homeodomain sequence as identified by the SMART domain prediction [48] (Figure 2).

The genomic organization of the *Mnemiopsis *Lhx genes showed no apparent linkage groups and they were found to be located on separate genomic scaffolds. The size of the scaffolds is as follows: *MlLhx1/5 *is found on scaffold ML1325 with a length of 127,187 bases. *MlLmx *is found on scaffold ML0569 with a length of 734,417 bases. *MlIslet *is found on scaffold ML0530 with a length of 126,527 bases. *MlLhx3/4 *is found on scaffold ML0681 with a length of 490,590 bases.

**Figure 3:**
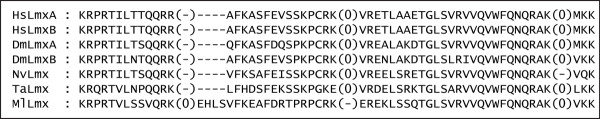
**Alignment of Lmx homeodomain sequence**. Alignment of the *Lmx *genes homeodomain region, showing the conservation of two intervening introns labeled (0). *Mnemiopsis*'s first intron position is 3' to the highly conserved position in other taxa. The second intron position is shared with other taxa, but not with the anthozoan *Nematostella*. Dm,*Drosophila Melanogaster*; Hs,*Homo sapiens*; Ml, *Mnemiopsis leidyi*; Nv,*Nematostella vectensis*; Ta,*Trichoplax adhaerens*.

In bilaterians, there are two conserved introns in the *Lmx *genes, within the homeodomain sequence. Both conserved intronic breaks are present in *Trichoplax *[6] a non-bilaterian species; however, the first intron position is slightly translocated in *Mnemiopsis *(Figure 3), and the second intron, while conserved in many other taxa, is not conserved in *Nematostella*. The homeobox sequences of *Lhx1/5, Lhx3/4*, and *Islet *were also surveyed for conserved intron positions (Additional file 1). Although introns were found within the homeodomain sequences of *Lhx1/5, Lhx3/4*, and *Islet*, they did not appear to have any conservation with any of the other taxa sampled.

### Phylogenetic relationships

**Figure 4:**
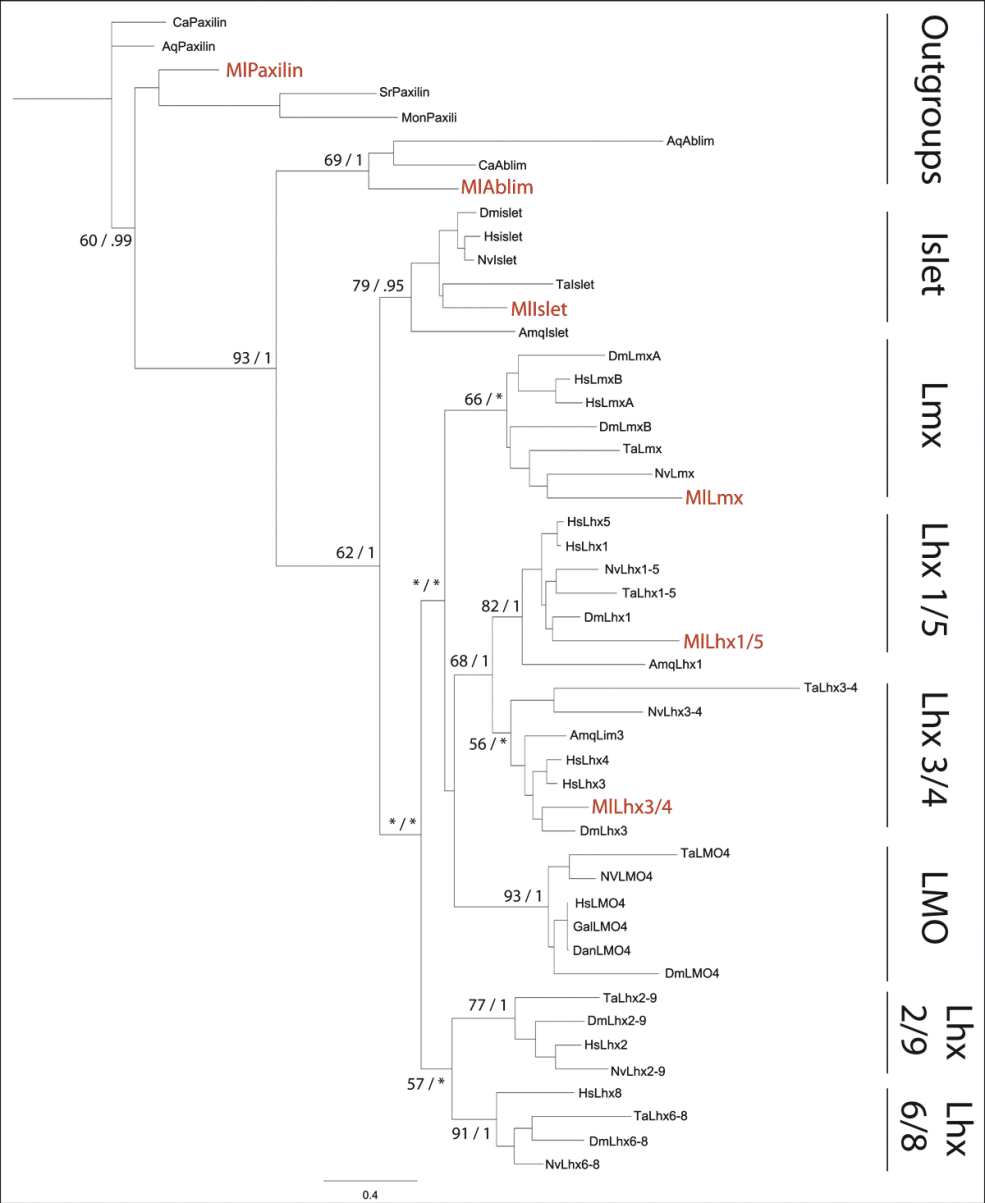
**Phylogenetic Tree of Lhx genes**. Maximum likelihood and Bayesian analysis consensus tree based on the tandem LIM domains and homeodomain, support values are indicated by Likelihood > 50/Bayesian analyses >.95, low support indicated by an asterisk. Construction of the Lhx gene orthology was conducted in RaxML v7.0.0 [82] using maximum likelihood analysis under the JTT I+G model of evolution determined by ProtTest v1.4 analysis [83]. A total of 1,000 searches was performed and 500 bootstrap replicates were applied to the tree with the best likelihood score used to generate branch support values. Bayesian analysis used two runs of Mr Bayes after 2,000,000 generations, JTT I+G, burnin = 5000 trees. Tree was rooted to the unicellular choanoflagellate *Monosiga brevicollis *LIM domain containing gene paxilin. Aq, *Amphimedon queenslandica; Ca, Capsaspora owczarzaki; *Dan, *Danio rerio; *Dm, *Drosophila melanogaster; *Gal, *Gallus *gallus; Hs, *Homo sapiens*;, Ml, *Mnemiopsis leidyi; *Mon, *Monosiga brevicollis; *Nv, *Nematostella vectensis*; Ta,*Trichoplax adhaerens*.

Phylogenetic analyses of the isolated ctenophore Lim homeodomain-containing genes were performed using sequence data from the Lhx complement of the cnidarian *N. vectensis *[49], the placozoan *Trichoplax adherens *[6], the sponge. *A. queenslandica *[50], and from the published genomes of *Homo sapiens, Danio rerio, Gallus gallus *and *Drosophila melanogaster*. Maximum likelihood and Bayesian analysis were performed individually on the aligned tandem LIM domains and the homeodomain (Figure 4). Similar to analyses using only the homeodomains [45], our analyses show relatively high support for the four *Mnemiopsis *Lhx genes within the *Lhx1/5, Lhx3/4, Islet*, and *Lmx *subclasses. The sponge *A. queenslandica *lacks an *Lmx *gene homologue, but due to low branching support between subclasses, we cannot determine whether the demosponge lineage lost the *Lmx *gene, or if this lineage diverged before *Lmx *arose. These predictions need to be tested with other sponge genomes.

Included in this phylogenetic study are three LIM domain-containing gene families that lack a homeodomain and are the most closely related to Lhx genes, which were used for outgrouping: nuclear LIM only (*LMO*), Actin binding LIM (*Ablim*), and *Paxilin*. The most closely related family to the Lhx genes are found within the nuclear LIM only (*LMO*) gene family [7]. Both *M. leidyi *and *A. queenslandica *do not possess genes within this family, and neither do the unicellular non-metazoans *Monosiga brevicollis *and *Capsaspora owczarzaki *surveyed in this study. The absence of *LMO *genes in these taxa in addition to the current genomic and phylogenetic data available and their internal branching within the Lhx hierarchy in our phylogenetic tree (although with low support values) suggests that these genes are likely a subfamily of Lhx genes and arose through a gene duplication shortly thereafter losing their homeodomains after the split of ctenophores and sponges and before the rise of ParaHoxozoa.

The four *M. leidyi *Lhx genes correspond closely to the *A. queenslandica *complement in gene content. In addition we also found that there are no homologues to the LIM-domain binding factors (*Ldb*) in the currently available sponge and ctenophore genomes, although this gene is present in all other animals. These data suggest that the ancestor of sponges and ctenophores contained three or four of the seven subclasses of Lhx genes, with Parahoxozoans acquiring the two closely related subclasses (*Lhx 2/9, Lhx 6/8*), *Ldb *factors, and the *LMO *gene family (derived from an already existing Lhx gene family) after their divergence from sponges and ctenophores (Table 1).

**Table 1:** Genomic complement of Lim homeobox genes

	Cnidaria *Nematostella*	Placozoa *Trichoplax*	Ctenophora *Mnemiopsis*	Porifera *Amphimedon*
Apterous *Lhx2/9*	L-L-H	L-L-H		
Arrowhead *Lhx6/8*	L-L-H	L-L		
*Islet*	L-L-H	L-L-H	L-L-H	L-L-H
*Lhx1/5*	L-L-H	L-L-H	L-L-H	L-L-H
*Lhx3/4*	L-L-H	L-L-H	L-L-H	L-L-H
*Lmx*	L-H	L-L-H	L-L-H	
*LMO*	L-L	L-L		
*Ldb*	LB	LB		

The genomic complement of LIM homeobox genes within the four basal metazoans. Ctenophores contain a genomic complement more similar to sponges than to placozoa and cnidarians, which have the full bilaterian complement of LIM homeobox genes. No synteny is found between the LIM homeobox genes in *Mnemiopsis*. H, Homeobox domain; L, LIM binding domains; LB, LIM binding domain.

### Developmental expression of *M. leidyi *Lhx genes

**Figure 5:**
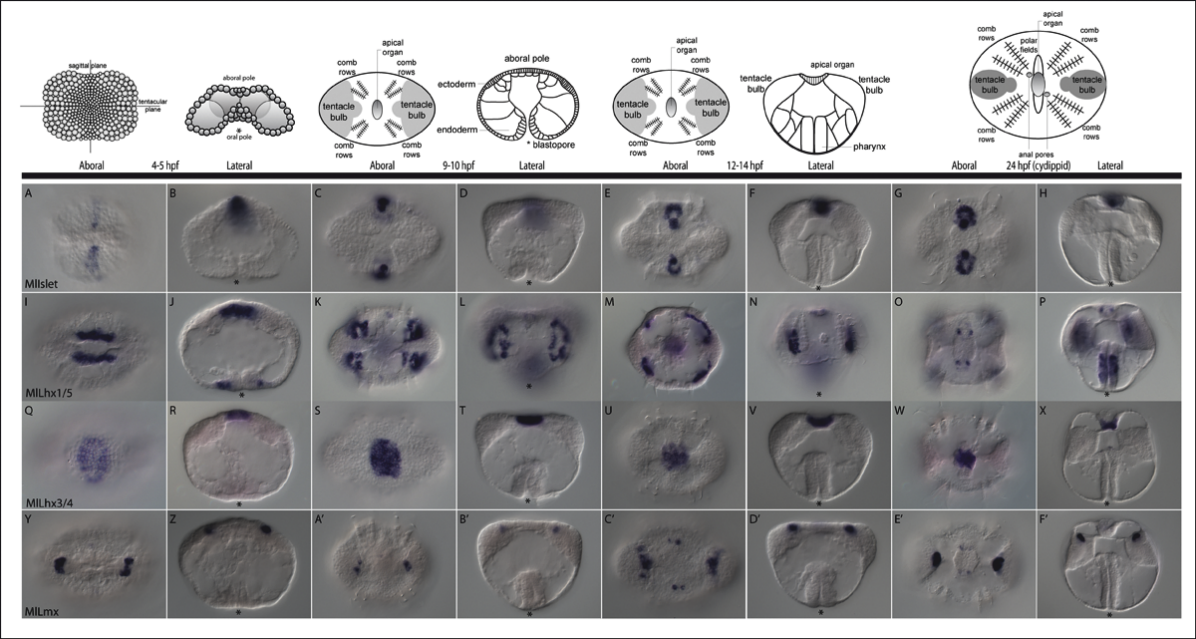
**Whole mount *in situ *hybridization of Lhx genes in *Mnemiopsis***. Whole mount *in situ *hybridization of *MlIslet *(**A-H**), *MlLhx1/5 *(**I-P**), *MlLhx3/4 *(**Q-X**), and *MlLmx *(**Y-F'**), purple staining represents the localization of detected mRNA transcripts. The above cartoons are representations of the underlying corresponding stages of development. All views are either aboral or lateral where indicated, with the asterisk representing the blastopore and future mouth. (A-H) *MlIslet *is expressed in aboral ectodermal cells, adjacent to *MlLhx3/4*, that give rise to part of the apical organ floor as well as the polar fields. (I-P) *MlLhx1/5 *is expressed around the blastopore, which gives rise to the pharynx. It is also expressed in mesodermal cells that give rise to the photocytes, which underlie four of the comb rows. There is late expression in four small groups of cells in the apical organ which overlap with cells that give rise to proposed photosensory cells. (Q-X) *MlLhx3/4 *is expressed in the aboral ectoderm, in a large group of cells that gives rise to part of the apical organ. (Y-F') *MlLmx *is expressed in two groups of cells that give rise to part of the tentacle bulb apparatus. Expression is also found in the same four groups of cells in the apical organ that overlap with the *MlLhx1/5 *expression.

To determine potential roles for Lhx genes in *M. leidyi *development, we looked at the spatial and temporal expression of all four Lhx genes through whole mount *in situ *hybridization. MlIslet is the earliest gene detected, at gastrulation or approximately four hours post-fertilization (hpf), in two to three rows of cells at the aboral pole along the sagittal axis (Figure 5A). This expression continues through development (Figure 5B-E), eventually forming the polar fields, as well as the most sagittal regions of the apical organ floor. The other Lhx genes are detected just after gastrulation, at approximately 5 hpf. *MlLhx1/5 *is expressed in a subset of mesodermal micromeres born at the oral pole (site of gastrulation) that enter the blastocoel, as well as in cells surrounding the blastopore (Figure 5I-J). These mesodermal oral micromere derivatives proliferate and migrate to areas underlying the comb plates (Figure 5K-N) to form the presumptive photocytes, or light-producing cells. The blastoporal expression continues through development, with expression later confined to individual cells of the pharynx. At the cydippid stage, there is an additional expression domain in the floor of the apical organ (Figure 5O-P). These four groups of cells possibly correspond to the putative photoreceptor cells [51].

*MlLhx3/4 *is expressed in cells at the aboral pole, eventually being confined to the central part of the apical organ floor (Figure 5Q-X). *MlLmx *is expressed in two groups of cells at the aboral pole just after gastrulation, along the tentacular plane (Figure 5Y). These cells eventually form part of the tentacle bulbs (Figure 5C'-F'). At 12 to 14 hpf, there is an additional expression domain in the ectoderm of the aboral pole in four groups of cells (Figure 5C', E'), which later appear to overlap with *MlLhx1/5 *in the region of the putative photoreceptors in the floor of the apical organ (Figure 5O).

**Figure 6:**
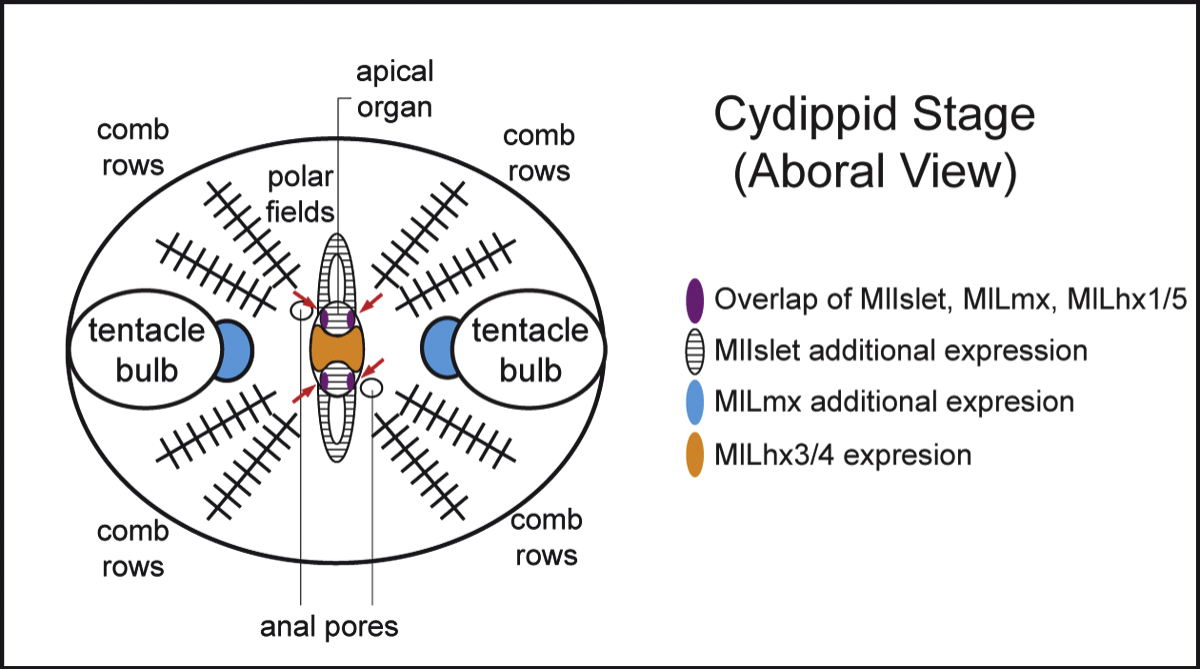
**Lhx expression diagram**. Summary diagram of the aboral overlapping and non-overlapping Lhx expression domains during the cydippid stage of *Mnemiopsis*. Overlapping expression is found in the four groups of cells within the apical organ that correspond to putative photosensory cells, indicated by red arrows. Non-overlapping domains include portions of the apical organ and associated polar fields, highly innervated sensory and nervous structures.

In summary, all four Lhx genes are expressed in the apical organ at the cydippid stage, in addition to other regions (Figure 6). *MlLhx1/5, MlLmx*, and *MlIslet *are expressed in an overlapping region, while *MlLhx3/4 *is expressed in other gene-specific regions of the apical organ. The other regions of expression include the polar fields (*MlIslet*), photocytes and pharynx (*MlLhx1/5*), and tentacle bulb apparatus (*MlLmx*).

## Discussion

### Lhx gene complements

By probing the full genome of *M. leidyi*, we find that it has a genetic complement more similar to that of the sponge lineage than to the complete repertoire of Lhx gene subfamilies found in placozoans, cnidarians, and bilaterians. These data provide further evidence of the basal position of ctenophore and sponge lineages relative to Parahoxazoans. While multiple independent gene loss in both ctenophore and sponge clades may be the cause for this apparent synapomorphy, growing arguments based on genomic content, are suggesting a close link between sponges and ctenophores, including the existence of homeodomain containing transcription factor complement [45], nuclear receptor repertoire [47], Wnt signaling pathway [46], and TGF beta pathway [52].

### *Mnemiopsis *expression data

Lhx genes are expressed in regions which contain the highest concentration of neural elements in the cnidarian *Nematostella*, including the apical tuft and nerve rings around the mouth and in the pharynx [6]. In the sponge *Amphimedon*, there are broader expression domains; however, there are overlapping domains associated with the photosensory pigment ring, suggesting that Lhx genes are playing a combinatorial role in neural or sensory cell fate specification across the Metazoa, including the basal metazoans [6]. The expression data here suggest Lhx genes are deployed in a similar fashion in the ctenophore *Mnemiopsis*. There are both overlapping and gene-specific expression domains associated with the apical sensory organ, a highly innervated structure involved in gravity, light, and pressure detection [53]. *MlLhx1/5, MlLmx *and *MlIslet *have overlapping expression in the putative photoreceptor cells (Figure 6), while *MlLhx3/4 *and *MlIslet *are also expressed in other parts of the apical organ. It is possible that a 'LIM code' is involved in specifying different neural territories or cell types of the apical organ. Although the molecular interactions of only *Lhx3/4 *and *Islet *have been studied in depth [22], it is not unlikely that other Lhx genes may be able to form complexes to differentially regulate gene expression patterns. The expression of Lhx genes also forms distinct boundaries within the apical organ and associated polar fields, which may contribute to regional specificity, axonal projection boundaries, or neural transmitter phenotypes of these neural structures as is seen in bilaterians [54].

Previous studies have described other genes in different regions of the apical organ of Mnemiopsis, including the T-box genes *Tbx2/3 *and *Brachyury *[55], the homeobox genes *Prd1 *and *Prd3 *[56], the Wnt genes *MlWnt6 *and *MlWntX *[46], as well as the zinc fingers *MlGli *and *MlGlis *[57]. Histological and electron microscopic studies of the ctenophore apical organ have suggested it is a highly innervated sensory structure [58-71]; however, gene expression studies suggest that it is much more complex than previously thought. These data are supported by recent work in the ctenophore, *Pleurobrachia pileus*; immunohistochemical studies have shown that there is a highly complex nerve net underlying the apical organ, as well as distinct groups of neurons within the apical organ floor that belie its morphological simplicity [72]. Using this information about the neural anatomy of ctenophores, we suggest that ctenophore Lhx genes are expressed in a combinatorial fashion in the developing sensory cells associated with the apical organ.

In addition to the apical organ, Lhx expression domains are also seen in putative neurosensory regions such as the polar fields expression of *MlIslet*. While these ciliated structures have been identified morphologically and histologically [59,72], their function has remained largely unknown. They have been proposed to be olfactory organs [58,73-75], however, there is little evidence to confirm this. The recent immunohistological study of *P. pileus *by Jager *et al*. [72] identified a special nerve net that extended throughout the polar fields, as well as extensive neuro-sensory structures, termed 'Z bodies.' The gene expression of *MlIslet *overlaps with the Z bodies, but is not specific to them; instead the expression domain encompasses the entirety of the polar fields.

The expression of *MlLhx1/5 *in the presumptive photocytes is also quite intriguing. Ctenophores are capable of bioluminescence via calcium-activated photoproteins [76], but the mechanism or function has yet to be elucidated. The photocytes are located around the meridional canals underlying the comb rows [77]. In addition to photoprotein genes, these cells also express an opsin gene [78], which suggests that these cells may represent an ancestral neuroeffector cell type. They express a gene that senses the environment (light-sensing via opsin), and an effector (photoprotein, generating light production) preceded by a transcription factor (*MlLhx1/5*), such that these photocytes appear to be capable of both sensing as well as responding to stimuli.

*MlLhx1/5 *expression is also found around the blastopore and is regionally localized to a portion of the pharynx in later stages. Considering that *Lhx1/5 *is expressed around the blastopore in many bilaterians, as well as the cnidarian *Nematostella*, we can speculate that besides neural patterning, *Lhx1/5 *was an ancestral 'blastoporal' gene. However, whether it functions in organizing activity in ctenophores, as it does in bilaterians, remains to be determined.

## Conclusion

Recent gene content studies [45-47] give support for Porifera and Ctenophora diverging prior to the ParaHoxozoa (Placozoa, Cnidaria, Bilaterian) during early animal evolution. This is further supported by the present study, in which we have further classified the genomic complement of *M. leidyi *Lhx genes. Comprehensive phylogenetic analyses of LIM genes [79] in addition to our Lhx gene phylogenetic analyses of cnidarians, ctenophores, sponges and placozoans, indicate that the ancestor of ctenophores and sponges likely had three or four Lhx gene subfamilies: *Lhx1/5, Islet, Lhx 3/4*, and *Lmx*. The common ancestor of ParaHoxozoa contained three more Lhx gene subclasses, the LIM domain binding proteins, and the gene family *LMO *which was formed by duplications from a pre-existing Lhx gene subclass. *Mnemiopsis *Lhx genes are expressed in both overlapping and non-overlapping domains within proposed neural and sensory cell territories. These data suggest that Lhx genes first likely played a role in the patterning of sensory cells, as is seen in sponge larval photoreceptive cells [6]. In other animal lineages with more complex nervous systems, Lhx genes appear to have a conserved role in patterning neural and sensory cell types. This apparent link shows support for the hypothesis that neural cell types may have evolved from non-neural sensory cells. An alternate hypothesis may be that sponges lost components of a simple nervous system after the split with ctenophores, or the ctenophore nervous system arose independently as a case of convergent evolution. These competing hypotheses still need to be considered due to the unexpected complex repertoire of structural and patterning nervous system genes found within sponges [34,35]. Future genomic, expression, and functional studies are needed to characterize the molecular nature of the ctenophore nervous system.

## Methods

### Animal collection, RNA extraction and cDNA synthesis

*M. leidyi *adults were collected from Eel Pond or the NOAA Rock Jetty in Woods Hole, MA, during the months of June and July and spawned as previously described [80]. RNA was extracted from embryos at regular intervals from fertilization to 36 hpf using TRI Reagent (Molecular Research Center, Cincinnati, OH, USA) [80]. RNA was reverse transcribed to generate cDNA using the SMARTer RACE cDNA Amplification Kit (BD Biosciences, San Jose, CA, USA).

### Identification of LIM homeobox genes in ctenophores

The *Mnemiopsis *genome was scanned in silico for genes of interest using a reciprocal BLAST approach. Human, *Drosophila*, and *Nematostella *orthologs were used as queries for TBLASTN searches. Candidate matches were then used in BLASTP searches of the human genome to find the closest hit. If the closest match was not the original ortholog or the E-value was greater than 0.001, then it was coded as being absent from the genome. For all genes of interest, gene-specific primers were designed for RACE PCR (MacVector, Cary, NC, USA). RACE PCR fragments were then conceptually spliced and aligned back to genomic contigs for comparison of intron-exon boundaries using Sequencher (Gene Codes, Ann Arbor, MI, USA). The following genes were isolated and fully sequenced: *MlLhx1/5 *[Genbank: JF912807], *MlLhx3/4 *[Genbank: JF912808], *MlIslet *[Genbank: JF912806], and *MlLmx *[Genbank: JF912809].

### Whole mount *in-situ *hybridization

Embryos were fixed at various stages from freshly collected uncleaved embryos (0 hpf) to cydippids (24 to 36 hpf) [80]. They were stored in methanol at -20°C until ready to use. Digoxygenin-labeled *M. leidyi *Lhx gene riboprobes (Ambion/Applied Biosystems, Inc., Foster City, CA, USA of the following sizes: *MlIslet *1200 bp, *MlLmx *1100 bp, *MlLhx1/5 *850 bp, and *MlLhx3/4 *1500 bp, were hybridized for 48 hours at 60°C at 0.1 ng/ul and detected using an alkaline phosphatase conjugated antibody (Roche Applied Science Inc, Indianapolis, IN, USA) and the colorimetric substrate NBT/BCIP (Roche Applied Science Inc, Indianapolis, IN, USA) Following detection, specimens were washed with phosphate-buffered saline and transferred through a glycerol series up to 70% glycerol. They were then mounted on glass slides, viewed under an Axioskop 2 compound microscope, and imaged using an AxioCam HRc with Axiovision software (Zeiss Inc, Jena, Germany). Color balance and brightness were adjusted using Adobe Photoshop CS3. The only modification to the *in situ *protocol is a change in acetic anhydride treatment (treated in 0.1 M triethanolamine rather than 1% w/v). For the most recently updated protocols, contact the authors. All *in situ *images presented here, as well as additional developmental stages and/or views, are available online via the comparative gene expression database, Kahikai http://www.kahikai.com.

### Phylogenetic analysis

*M. leidyi *Lhx protein sequences were aligned to the complements of Lhx proteins of the various species: *H. sapiens, Drosophila melanogaster, N. vectensis, T. adhaerens*, and *A. queenslandica*, and outgroup sequences of LIM-only domain containing gene 4 (*LMO4*) in addition to the above species when present, *G. gallus, D. rerio*, and the related unicellular choanoflagellates *M. brevicollis*, and *C. owczarzaki *LIM domain-containing genes *paxilin *and *ablim *(full sequence data provided in Additional file 2, using MUSCLE (default parameters) and trimmed by eye in Jalview [81] to include the conserved LIM domains and Homeodomains. Construction of the Lhx gene orthology was conducted in RaxML v7.0.0 [82], using maximum likelihood analysis under the JTT I+G model of evolution determined by ProtTest v1.4 analysis [83]. A total of 1,000 searches was performed and 500 bootstrap replicates were applied to the tree with the best likelihood score used to generate branch support values. Bayesian phylogenetic analyses were also performed with MrBayes 3.1 [84] using the JTT I+G model with two runs of 2,000,000 generations sampled every 100 generations. The first 5,000 trees were disregarded as burn-in.

## Abbreviations

Ablim: actin binding LIM; bp: base pair; BLAST: Basic Local Alignment Search Tool; CNS: central nervous system; hbf: hours post fertilization; HD: homeodomain; *Ldb*: LIM domain-binding protein; Lhx: LIM homeobox containing genes; LMO: LIM-only domain containing gene; LMO4: LIM-only domain containing gene 4; ML: maximum likelihood; ParaHoxozoa: Placozoa: Cnidaria: Bilateria; PCR: polymerase chain reaction; RAxML: randomized accelerated ML.

## Competing interests

The authors declare that they have no competing interests.

## Authors' contributions

DS drafted the manuscript, performed the sequence alignment, and performed the phylogenetic analyses. DS and KP performed the animal collection, gene isolation, sequencing and *in situ *hybridizations. KP helped in drafting the manuscript and in the conception of the study. MQM participated in the design and conception of the study and helped to draft the manuscript. All authors read and approved the final manuscript.

## Supplementary Material

Additional file 1 **Alignments of Lhx gene homeodomain sequences**. Alignment of *Lhx1/5, Lhx3/4*, and *Islet *genes homeodomain regions. Introns positions indicated by (0). Amq, *Amphimedon queenslandica*; Hs, *Homo sapiens*; Ml,*Mnemiopsis leidyi; *Nv, *Nematostella vectensis*; Ta, *Trichoplax adhaerens*. A. Human *Lhx1 *and *Lhx5 *have a shared intron not found within any other species. The *Mnemiopsis Lhx1/5 *gene contains an intron that is also not shared with any other species in this study and appears to be species specific. B. The *Lhx3/4 *gene in *Mnemiopsis *has two introns not found in any other species examined and appears to be species specific. Humans also have an intron position not shared with the other species. C. The *Islet *gene in *Mnemiopsis *has a species specific intron position interrupting its homeodomain sequence. *Nematostellla *has an intron interrupting the homeodomain sequence in a different location than the ctenophore sequence. Both the *Mnemiopsis *and *Nematostellla *intron positions do not overlap and seem to not be related. Humans, placozoans, and sponges do not have introns interrupting the homeodomain sequences. Click here for file

Additional file 2 **Full sequence data**. The full sequence data of all of the LIM genes used in this study. Gene accession numbers, corresponding gene names used in the phylogeny, and species name are listed in table format. Click here for file
