# Supplementary figures and images for: Lim homeobox genes in the Ctenophore Mnemiopsis leidyi: the evolution of neural cell type specification
Source: EvoDevo. 2012 Jan 13;3:2. doi: 10.1186/2041-9139-3-2 (PMC3283466; doi:10.1186/2041-9139-3-2)

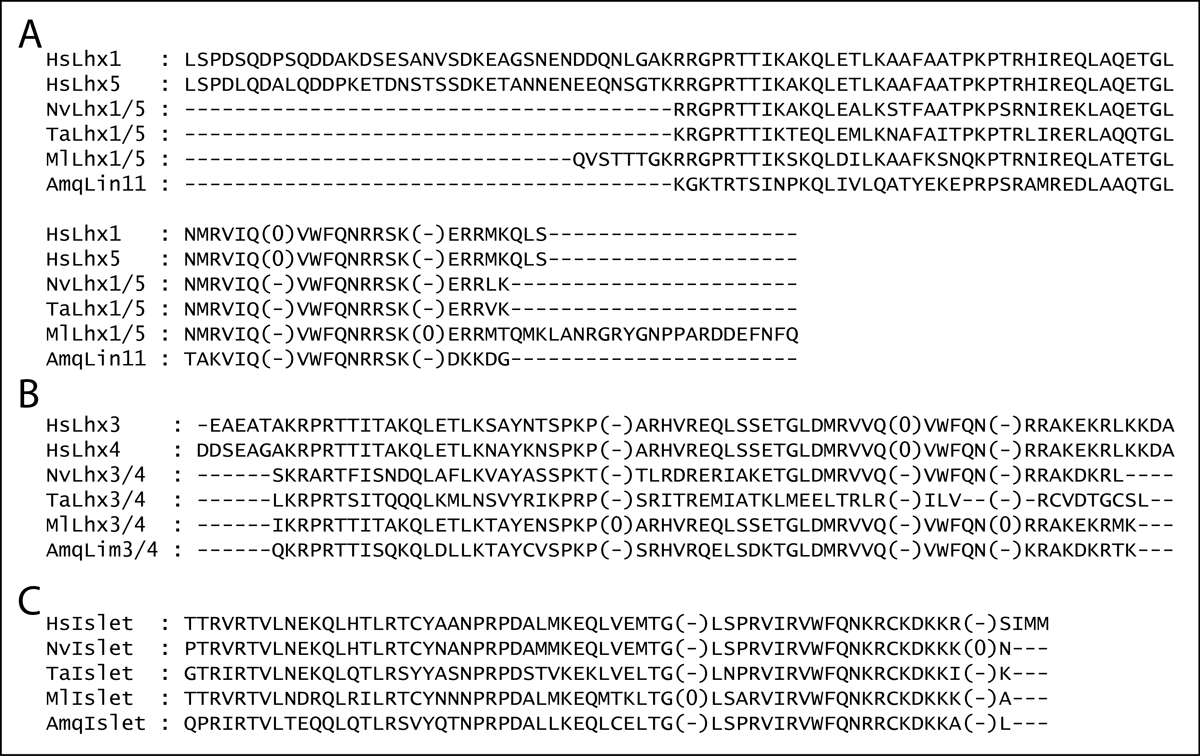

Supplement: Additional file 1 — Alignments of Lhx gene homeodomain sequences. Alignment of Lhx1/5, Lhx3/4, and Islet genes homeodomain regions. Introns positions indicated by (0). Amq, Amphimedon queenslandica; Hs, Homo sapiens; Ml,Mnemiopsis leidyi; Nv, Nematostella vectensis; Ta, Trichoplax adhaerens. A. Human Lhx1 and Lhx5 have a shared intron not found within any other species. The Mnemiopsis Lhx1/5 gene contains an intron that is also not shared with any other species in this study and appears to be species specific. B. The Lhx3/4 gene in Mnemiopsis has two introns not found in any other species examined and appears to be species specific. Humans also have an intron position not shared with the other species. C. The Islet gene in Mnemiopsis has a species specific intron position interrupting its homeodomain sequence. Nematostellla has an intron interrupting the homeodomain sequence in a different location than the ctenophore sequence. Both the Mnemiopsis and Nematostellla intron positions do not overlap and seem to not be related. Humans, placozoans, and sponges do not have introns interrupting the homeodomain sequences. [file 2041-9139-3-2-S1.PNG]
